# A cross-sectional study of personality traits in women previously treated or untreated for alcohol use disorders

**DOI:** 10.1186/1747-597X-2-24

**Published:** 2007-08-06

**Authors:** Anette Östlund, Gunnel Hensing, Annika Jakobsson, Valter Sundh, Fredrik Spak

**Affiliations:** 1 The Sahlgrenska Academy at Goteborg University, Department of Public Health and Community Medicine, PO Box 453, SE-405 30 Göteborg, Sweden

## Abstract

**Background:**

A better understanding of the relationship between treatment-seeking for alcohol problems and personality traits could give useful insight in factors promoting or hindering treatment for alcohol use disorders (AUD). The aim of this study was to analyze the associations between treatment-seeking for AUD, personality traits, and psychiatric co-morbidity in women. The study was based on pooled cross-sectional data from three population based samples and one clinical sample (n = 1,339). Comparisons were made between treated and untreated women with AUD, and between those with resolved and unresolved AUD.

**Results:**

A stepwise logistic regression model showed that treatment-seeking for AUD was not associated with personality traits. Among women with lifetime AUD (n = 217), those who had been treated (n = 42) had significantly higher scores than untreated women (n = 175) on three personality traits of the Karolinska Scales of Personality (KSP); somatic anxiety, muscular tension, and guilt. Women with resolved AUD, who had received treatment (n = 23) had significantly higher scores on scales measuring somatic anxiety, psychic anxiety, muscular tension, irritability, and guilt than untreated women with resolved AUD. The latter group resembled women without AUD on most personality traits. There were no differences in occurrence of lifetime psychiatric disorders between the treated and the untreated women, whereas treated women with current AUD had increased risk of lifetime anxiety (OR: 3.1, 95% CI: 1.1–8.7).

**Conclusion:**

Treatment-seeking was not associated with personality traits in this study. Still, it can be concluded that women with resolved AUD who had received treatment had high scores on the KSP-scales measuring psychic and somatic anxiety, tension, irritability, and feelings of guilt. This suggests that personality assessment might be a useful tool in tailoring individual treatment programs for women with AUD. Future studies need to explore if women who do not seek treatment have special needs which are not met in usual treatment settings.

## Background

Treatment-seeking for alcohol use disorders (AUD) is a complex process that often stretches over months or years and can differ between women and men [1], cultures, social settings, severity of alcohol problems and the presence of psychiatric co-morbidity [2]. It is known that personality plays a role in the development of AUD [3,4], in treatment attrition [5], treatment outcome [6], and relapse [7], but its role in treatment-seeking has not previously been explored.

Personality traits are viewed merely as convenient summary labels for observed consistencies in behavior by some, while others assert that personality traits are fundamental causal variables and thus provide cogent explanations for consistencies in behavior [8]. In our study personality traits are regarded as independent contributing factors in the development of alcoholism and thus potentially also important for treatment-seeking behaviour.

The treatment-seeking process in people with alcohol problems has been found to be fragile and the personal willingness to change appears to be vitally important [9]. Studies have shown that high severity of drinking and social problems promote the decision to seek treatment [10], as does the experience of an interpersonal crisis, problems in social/personal relations, or pressure from significant others [11]. Additional psychiatric diagnoses [12], an earlier treatment history and unemployment [13], have been reported to increase the likelihood of seeking treatment. Mood and anxiety disorders have been found to precede substance abuse problems in women [14] and when seeking alcohol treatment, women often identify anxiety, depression, and stressful life events as their main problems [15].

The majority of individuals with drinking problems remain untreated [16] and at least half of all remissions from alcohol abuse or dependence occur without specific treatment given [17]. Individuals recovering with the help of treatment have been reported to, previous to treatment, have had more severe problems over longer periods of time compared with individuals recovering without treatment [17].

Associations between personality and mental health service utilization have been established; conscientiousness was found to be associated with decreased likelihood of mental health service use, while neuroticism was associated with increased use of the services [18]. Personality may also act as mediator in treatment-seeking for AUD in women, and a better understanding of the relationship between treatment-seeking and personality traits could add useful knowledge to the complex treatment-seeking process. To our knowledge, no previous study has addressed this specific subject. The aim of this study was to analyze the associations between treatment-seeking for AUD, personality traits and psychiatric co-morbidity in women.

## Methods

### Study sample and study design

The study was done within the longitudinal project titled, "Women and alcohol in Göteborg" (WAG) [19]. The WAG-data is collected as a two phase technique epidemiologic study with an initial screening phase followed by individual face to face interviews (Table 1). The procedures are in concordance with recommendations for studies of low-prevalence diseases [20]. The project was carried out in one urban (93 157 inhabitants) and one suburban (105 683 inhabitants) district, in Göteborg, which is the second largest town in Sweden, with a total of 444 553 inhabitants 1995 [21]. Compared to Göteborg at large the population in the uptake area was younger, healthier with higher incomes, but in most socio-demographic variables it was similar to national rates of Sweden [19]. The study sample also includes a clinical sample consisting of consecutive patients visiting all relevant public medical service units in the uptake area; including three mother care units, seven general practice centers, the three emergency wards for internal medicine, surgery and general psychiatry as well as two psychiatric outpatients units.

**Table 1:** Study design of the project Women and Alcohol in Göteborg, Sweden, and the study group of the personality and treatment seeking study.

**Sampling procedure**	**Clinical sample n**	**General population sample n**
Phase 1: selected for screening	Consecutive patients in public medical service N = 2,852	Birth cohorts; women born 1925, 1935, 1945, 1955, 1965, 1970, 1975, and 1980 N = 7,143
Participation	2,154 (76 %)	5,509 (77 %)
Phase 2: selected for interviewing	412	1,845
Face to face interview	222	1,428
Completed the KSP* and the interview	171 (77% of interviewed)	1,168 (82% of interviewed)

**Final study group** **Clinical sample + General population sample n = 1,339**		

* Karolinska Scales of Personality (KSP)

### Screening phase

As a first step in the screening phase, a 13-item questionnaire developed for screening of alcohol problems (Screening, Women and Alcohol in Göteborg, SWAG) was sent, in 1986, to all women born in 1925, 1935, 1945, 1955, and 1965 who were identified in the official population register for District West (population 99 328) of Göteborg on December 31, 1985. The 13 items questionnaire was constructed as a Likert scale with a maximum score of 13 points (indicating alcohol related problems). When validated for screening of female AUD, sensitivity was > 74 %, specificity > 96 % and PPV 40–50 %, which was regarded as satisfactory. A more detailed description of the psychometric properties of SWAG can be found elsewhere [19]. Additional birth cohorts (women born in 1970/1975 and 1980) were included in 1995 and 2000, employing the same procedures as in 1986. The screening procedure was the same for the clinical sample, with the exception that the screening questionnaires were distributed during visits to the medical service units. For the analyses, women > 70 years of age were excluded from the clinical sample, since no corresponding age group existed in the population based samples.

### Interview phase

After screening, a stratified random sample was selected for interviewing. The stratification groups were made up of all respondents with a score of ≥ 4 points on SWAG, and respondents with a score of 1–3 points, 0 points, and drop outs in the screening phase. The three latter groups were selected with varying percentages in order to form groups of similar sizes. Interviews were done face to face in 1990, 1995, and 2000 respectively. The semi-structured interviews comprised approximately 1000 items and took from 1 1/2 up to several hours to perform. The interview focused on socio-demographic characteristics, alcohol consumption, health issues and included questions about experiences of treatment for AUD. Treatment was defined as seeking treatment specifically for alcohol problems, and refers to any kind of treatment available. The interviewers were clinicians trained in making diagnoses according to the DSM-III-R, but only Axis I, IV and V diagnoses were made. The interviewers were trained until interrater agreement was reached at a diagnostic level, but not necessarily with regard to severity/remission/subtype specifiers.

The present study was based on cross-sectional data from 1990 (clinical and population samples), 1995 and 2000 (population samples). Although the clinical sample was recruited from services used by women representing the population at large, it differed from the population sample in some respects. The women were older, had more children, and slightly fewer were married/cohabiting. More women in the clinical population belonged to the lower social class, and fewer to the upper class. Furthermore, the symptom severity among women with AUD, calculated as the numbers of alcohol related symptoms multiplied with the duration in years, was higher in the clinical sample. There was no difference in educational level, measured as < 10 years of education (Table 2).

**Table 2:** Comparison of severity of alcohol problems and demographic characteristics in the clinical sample vs. the general population sample from 1990, analyzed by logistic regression, adjusted for age.

Variables	Clinical sample (n = 115)		
	
	n	OR (95% CI)	P-values
Severity^1^	508	1.5 (1.1–1.9)	0.004
Education (< 10 years)	497	1.1 (0.7–1.6)	0.778
Social class^2^	409	0.6 (0.4–0.9)	0.004
Number of children^3^	474	1.3 (1.0–1.7)	0.040
Married/cohabiting	495	0.7 (0.5–1.0)	0.049
Separated/single	495	1.3 (0.8–2.0)	0.220

Reference population = general population sample excluding persons with a diagnosis of alcohol use disorders (AUD). OR = odds ratio.

1. Number of alcohol related symptoms multiplied by the duration of each symptom in years. Three categories are used, 0 points, 1–19 points, and ≥ 20 points.

2. Three categories are used, lower, middle, and upper class. 3. Three categories are used, no children, one child, and two or more children.

We concluded that the differences between the samples would not in any important way interfere with the analysis of predictors for the outcome variables used in this study. Thus we pooled the samples in order to reach enough power in the statistical analysis. The numbers of women diagnosed with AUD needed for analyzing associations of personality traits and treatment-seeking were estimated to approximately 200.

Informed consent was obtained from the interviewees and the procedure was approved by the Research Committee for Ethics at the Faculty of Medicine at Göteborg University (Ö 591–99).

### Diagnoses

Lifetime psychiatric diagnosis were made with the *Diagnostic and Statistical Manual of Mental Disorders*, 3^rd ^d edition (DSM-III – R) [22]. After information obtained with the Composite International Diagnostic Instrument-Substance Abuse Module (CIDI-SAM) and/or from patient records, 2 diagnoses were added and 32 were excluded [19]. The diagnosis "lifetime AUD" refers to AUD occurring at any time in life (including also adolescence). In some computations, this group is divided into "current AUD" (= AUD during the last 12 months prior to the interview) and "resolved AUD" (= AUD present only before the last 12 months prior to interview). Other drug abuse is not included in the computations, mainly because the illicit drug use is very low in this study and other Swedish studies compared to the use of alcohol.

Diagnoses of anxiety and depressive disorders were likewise made by the interviewers according to the DSM-III-R. We constructed the dummy variables depression and/or anxiety in order to include all diagnoses we considered relevant to each condition and also to increase cell sizes [23]. Lifetime anxiety and lifetime depression refers to these diagnoses regardless of when in life they have occurred (including also childhood).

### Instruments

The Karolinska Scales of Personality (KSP) is a personality test originally constructed to measure aspects of personality closely linked to the information processing and arousal systems in the individual in order to find biological correlates of personality traits relevant in psychiatric research [24]. Thus this personality test is not intended to give a full description of personality. KSP consists of 135 statements grouped into 15 different scales. Each statement has four response alternatives: "disagree completely", "partly disagree", "partly agree" and "agree completely", with a score of 1, 2, 3 and 4 points, respectively. Some items are reversed so that "disagree completely" gives 4 points instead of 1. The points for each scale are then added up and the raw scores are transformed into T-scores. High scores indicate pathology on all scales except for the Socialization scale [24]. The scales included are described in Table 3. The stability of personality traits in the general population sample was tested over 5 years. Correlations between initial assessment and follow-up were high for most KSP scales (r = 0.48 – 0.77), with most values > 0.7, indicating individual stability. The strength of the correlations increased significantly with age [25].

**Table 3:** The Karolinska Scales of Personality (KSP) and descriptions of high scores for each scale.

Related personality traits	KSP scales	Description of high scores
Anxiety	Somatic anxiety	Autonomic disturbances, restless, panicky
	Psychic anxiety	Worrying, anticipating, lacking self-confidence
	Muscular tension	Tense and stiff, not relaxed
	Psychastenia	Easily fatigued, feeling uneasy when urged to speed up
	Inhibition of aggression	Lacks ability to speak up and be self-assertive in social situations
Extraversion-introversion	Impulsiveness	Acting on the spur of the moment, non-planning, impulsive
	Monotony avoidance	Avoiding routine, need for change and action
	Detachment	Avoiding involvement with others, being withdrawn
Conformity-non-conformity	Socialization	Positive childhood experiences, satisfied with present life situation
	Social desirability	Socially conforming, friendly, helpful *or *"faking good"
Aggression	Verbal aggression	Getting into arguments, berating people when annoyed
	Indirect aggression	Sulking, slamming doors when angry
	Irritability	Irritable, lacking patience
	Guilt	Remorseful, ashamed of bad thoughts
	Suspicion	Suspicious, distrusting people's motives

Descriptions reprinted with kind permission from Petter Gustavsson.

### Attrition in the study sample

The most common reasons given for not participating in the study were lack of time and low alcohol consumption (in the latter case with the assumption that their responses thus would be of little interest to the researchers). Another reason for attrition was that some of the selected women could not be reached by letter or telephone. A random quarter of the attrition group from the screening phase was invited to the interview phase. They did not differ significantly from those who participated in both the screening phase and the interview phase regarding alcohol consumption and prevalence of AUD, age and education [26]. Furthermore, we have fairly good knowledge about internal attrition. Most of the women in this group agreed to take part in a shorter telephone interview, and to these KSP was not administered.

In an earlier study within the WAG project medical files were searched, and as only four new cases were identified in the attrition group, this did not affect prevalence of AUD [19]. Thus it seems unlikely that there would be a significant overrepresentation of women with alcohol problems in the attrition group. However, if this had actually been the case, it would have implied that the differences we found were underestimated, not overestimated. Hence, the attrition does not appear to constitute a serious problem in interpreting the findings.

### Statistics

The reference group (1,122) included women who participated with a complete interview, and excluded women with AUD.

We report data as T-scores (transformed scores with a mean of 50 and one standard deviation for the reference population). The KSP is sensitive to age and educational level. We therefore used a linear regression model to estimate the effect of these factors, and added a quadratic model when this improved the fit of the model significantly. The raw scores were then adjusted for age and education and thereafter transformed into T-scores.

Significance tests for comparing two groups regarding personality scores were made with two-sample t-test. P-values < 0.05 are considered statistically significant, and 95% confidence intervals (CI) are given. When comparing two groups regarding occurrence of characteristics, e.g. diagnosis of anxiety, odds ratio with 95% confidence interval were calculated from two-by-two contingency tables.

Multi-variable analyses of factors related to treatment-seeking were performed with logistic regression models within each of the 15 scales using factors such as age, education, social class, and additional diagnoses of anxiety and/or depression as covariates.

## Results

### Personality in relation to treatment-seeking

Personality traits did not predict treatment-seeking for alcohol problems in a stepwise logistic regression model, after adjustment for age, education, social class, psychiatric co-morbidity, and severity. Women with lifetime AUD (n = 217) differed from women without AUD (n = 1,122) on twelve of fifteen scales of KSP. P-values estimated by two-sample t-test ranged from 0.001 to 0.0016. There were no significant differences in three scales: inhibition of aggression, detachment and social desirability (Table 4).

**Table 4:** Overview of personality traits in treated and untreated women diagnosed with AUD. Total sample n = 1,339, reference population without AUD n = 1,122.

	**Personality traits**	**Life time psychiatric diagnoses DSM-III-R n = 217**	**Treated/untreated women with lifetime AUD n = 217**	**Personality traits**	**Treated/untreated women with lifetime AUD Resolved n = 123**	**Personality traits**
**Women diagnosed with lifetime AUD n = 217 **†	↑impulsiveness ↑somatic anxiety ↑psychic anxiety ↑tension ↑psychastenia ↑monotony avoidance ↑aggression ↑guilt ↑suspicion ↑verbal aggression ↑indirect aggression ↓socialization	Anxiety 36 % Depression 21 %	**Treated n = 42 **††	↑somatic anxiety ↑tension ↑guilt	**Treated n = 23 **†††	↑somatic anxiety ↑psychic anxiety ↑irritability ↑tension ↑guilt
			
			**Untreated n = 175**	Resembles the reference population	**Untreated n= 100 **††††	↑somatic anxiety ↑psychic anxiety ↑monotony avoidance ↑psychastenia ↑impulsiveness ↑irritability ↓socialization

† = significant differences between the reference population (n = 1,122) and women diagnosed with lifetime AUD (n = 217).

††= significant differences between treated (n = 42) and untreated (n = 175) women with AUD.

†††= significant differences between treated (n = 23) and untreated (n = 100) women with resolved AUD.

†††= significant differences between untreated women with resolved AUD (n = 100) and the reference population (n = 1,122).

Among women with lifetime AUD (n = 217), the treated women (n = 42) had significantly higher scores than untreated women (n = 175) on three scales of the KSP; somatic anxiety, muscular tension, and guilt. The observed differences in the other scales were non-significant (Table 5).

**Table 5:** Treated and untreated women diagnosed with life-time alcohol use disorders (AUD). Personality traits measured with the Karolinska Scales of Personality (KSP) in a pooled sample (n = 1,339) reported as T-scores (transformed scores with a mean of 50) and 95% confidence intervals (CI).

	**Women with lifetime AUD Untreated (n = 175)**		**Women with lifetime AUD Treated (n = 42)**	
	**Mean**	**CI**	**Mean**	**CI**

Somatic anxiety*	**55.5**	**53.7–57.4**	**60.3**	**56.0–64.7**
**Psychic anxiety**	52.0	50.4–53.5	55.0	51.0–59.1
Muscular tension*	**53.2**	**51.4–54.9**	**57.8**	**53.5–62.2**
**Psychastenia**	52.2	50.6–53.7	54.8	51.4–58.2
**Inhibition of aggression**	49.1	47.7–50.6	49.7	46.6–52.8
**Impulsiveness**	54.6	53.0–56.2	55.2	51.9–58.5
**Monotony avoidance**	53.2	51.6–54.9	53.0	48.7–57.2
**Detachment**	51.5	49.9–53.1	51.3	47.8–54.8
**Socialization**	40.8	38.8–42.7	38.1	33.7–42.4
**Social desirability**	49.4	48.0–50.9	52.9	48.7–57.0
**Verbal aggression**	54.1	52.4–55.7	55.9	52.9–58.8
**Indirect aggression**	53.8	52.4–55.2	54.0	51.2–56.8
**Irritability**	53.5	51.9–55.2	56.6	53.3–59.8
Guilt**	**51.4**	**49.8–53.0**	**56.4**	**53.2–59.5**
**Suspicion**	52.4	50.6–54.1	55.1	51.6–58.7

*p < 0.05, **p < 0.01, (two sample t-test, df = 215)

More than half of the women with lifetime AUD had resolved their problems (n = 123), meaning that AUD was not identified during 12 months prior to the interview. Of those 19 % (n = 23) had received treatment. The treated women scored significantly higher than the untreated on somatic and psychic anxiety, irritability, tension, and guilt feelings (Table 6). The observed group differences (non-significant) in the other scales were in the expected directions. Compared with the treated women, the untreated women (n = 100) scored more similarly to the reference group (n = 1,122). However, they had significantly higher scores than the reference group on somatic and psychic anxiety, irritability, monotony avoidance, impulsivity and psychastenia scales, and lower scores on the socialization scale. There were no statistically significant differences between treated and untreated women with current AUD.

**Table 6:** Untreated and treated women with resolved alcohol use disorders (AUD). Personality traits measured with the Karolinska Scales of Personality (KSP) in a pooled sample (N = 1,339) reported as T-scores (transformed scores with a mean of 50 and one standard deviation for the reference population) and 95% confidence intervals (CI).

	**Women with resolved AUD Untreated (n = 100)**		**Women with resolved AUD Treated (n = 23)**	
	**Mean**	**CI**	**Mean**	**CI**

Somatic anxiety*	**53.7**	**51.3–56.1**	**61.1**	**54.6–67.7**
Psychic anxiety*	**51.8**	**49.8–53.8**	**57.0**	**50.8–63.1**
Muscular tension*	**52.0**	**49.8–54.1**	**58.9**	**52.6–65.3**
**Psychastenia**	51.8	49.6–53.9	55.7	51.4–59.9
**Inhibition of aggression**	50.7	48.8–52.6	51.4	47.3–55.5
**Impulsiveness**	53.9	51.8–56.1	56.1	51.4–60.9
**Monotony avoidance**	52.1	50.2–54.0	54.8	48.6–60.9
**Detachment**	51.3	49.3–53.4	51.1	46.3–55.9
**Socialization**	40.9	38.4–43.4	39.9	33.9–45.8
**Social desirability**	50.6	48.5–52.6	53.8	48.2–59.4
**Verbal aggression**	51.7	49.7–53.7	54.8	50.2–59.3
**Indirect aggression**	51.8	50.0–53.6	53.7	49.4–58.1
Irritability*	**52.4**	**50.3–52.7**	**57.6**	**52.7–62.4**
Guilt*	**50.6**	**48.5–52.7**	**57.5**	**52.5–62.5**
**Suspicion**	52.1	49.8–54.4	54.1	49.1–59.1

*p < 0.05, (two sample t-test, df = 121)

### Psychiatric co-morbidity in relation to treatment-seeking and personality

In women with lifetime AUD, 36% (n = 79) also had lifetime anxiety and 21% (n = 45) had lifetime depression. The corresponding figures in the sample without AUD, were 18% (lifetime anxiety) and 11 % (lifetime depression). Among women with resolved AUD, there were no differences in occurrence of psychiatric disorders (lifetime) between the treated (n = 23) and the untreated women (n = 100).

Among women with unresolved AUD we estimated an OR: 3.1 (95% CI: 1.1 – 8.7) for treated women with current AUD (n = 19), regarding the risk of having lifetime anxiety, compared to the untreated group (n = 75). There were no differences between the groups regarding lifetime depression, OR: 0.98 (95% CI: 0.3 – 3.4).

## Discussion

This study showed no significant associations between treatment-seeking and personality traits, but one of the main findings was that women with resolved AUD, who had received treatment, had higher scores on most KSP-scales compared to untreated women with resolved AUD. The latter was statistically significant for five KSP-scales: somatic anxiety, psychic anxiety, muscular tension, irritability and guilt. The scores differed most in women with lifetime AUD who also had other psychiatric diagnoses and had received treatment, while women who had resolved their AUD without treatment resembled the reference population. An explanation to our results could be that the subgroup of women with more anxiety related personality traits use alcohol as self-medication, and that these women need treatment to recover, while less anxiety prone women with AUD more readily manage to recover without assistance. Other studies have shown that persons with AUD in clinical samples, i.e. in treatment, show more anxiety compared to persons with AUD in general population samples [27].

According to the WHO/ISBRA study, the most important factor to contribute to a DSM-IV diagnosis of dependence was anxiety experienced by an individual on stopping to drink [28]. Anxiety has also been shown to predict relapse [29]. According to another study, women with alcoholism were more anxious, and felt more inferior, compared to women without alcoholism [30].

The highest KSP scores were found in women with the combination of lifetime AUD and other psychiatric diagnoses who had attended treatment for AUD. They worried more and were more tense, irritable, and restless than untreated women with comparable diagnoses. A similar personality pattern was found in women with depression reported in a study where early-onset major depression predicted more deviating scores than late-onset major depression [31]. The results imply that certain personality traits as well as psychiatric co-morbidity are important factors prompting women to seek help, but as ours is a cross-sectional study, it is not possible to draw conclusions on the causal relations.

### Methodological limitations and considerations

To our knowledge, no study has previously examined treatment-seeking for AUD in relation to personality traits. One of the advantages of this study was that the data stem from a population based sample, which gave us the opportunity to study women that will never be found in a clinical sample, as well as women who had entered treatment. A two-staged sampling was used since the prevalence of AUD among women is low and the prevalence of treatment-seeking is even lower.

The differences between the treated and untreated women with AUD, adjusted for age and education, are statistically significant for three of the 15 personality scales, which proves that the obtained sample size of about 200 cases, gives sufficient power to detect important associations between treatment-seeking and personality factors. The scale with the largest difference, guilt feelings, showed a mean difference between the two groups of 5 units unadjusted (p < 0.01), but 2.5 adjusted (p = 0.32). Similar relations were found for the other two scales with significant difference unadjusted. We think that the obvious interpretation of this result is that personality factors per se only play a small role in predicting treatment-seeking after controlling for the most important external factors. However, any difference will be statistically significant if the sample size is large enough, so for didactic reasons we performed a simulation study, using a bootstrap technique, to examine at what sample size we could expect to reach 5 % significance with probability 80 % in a multivariate binary logistic model for the three personality factors that were closest to be significant in the models with age, education, severity, psychiatric diagnosis and socio-economic status as covariates. Required sample sizes were approximately 450, 600 and 650, a result which we think agrees with our conclusion that low power is not a problem in this analysis.

In a 5 -year follow-up study personality traits in general were found to be stable among adult women [32]. Still, we cannot disregard the possibility that psychiatric disorders can influence personality traits and vice versa.

Pooling a population sample and a clinical sample was needed in order to obtain a sufficient sample size, but this procedure also created limitations of the study. Although the initial comparisons of the samples showed that sufficient similarities for the present analyses existed, the samples differed somewhat. For example did the women in the clinical sample score higher on anxiety, lower on socialization and had higher symptom severity. This may have influenced the results to some extent, but the clinical sample (n = 171) was small compared with the general population samples (n = 1,168), and it is mainly composed of women having attended GP's and maternity care units. These women do not differ greatly from the general population, as most women attend these units in Sweden. The conclusion is that this sample is fairly representative of at least Caucasian women in the Northern Europe.

The way in which personality traits interact with treatment requirements in relation to other factors needs to be studied further. The question remains whether women who never seek treatment should be identified and offered help. We do not think that the high percentage of all the many women who resolved their AUD without help should be interpreted as they did not need or would have benefited from help. These individuals experience difficult problems regarding alcohol (as the diagnoses indicates), and it is always important to try to reduce harm [33]. Thus, it is possible that the comparatively low proportion of women who resolve their AUD with the aid of treatment does not reflect the "full" treatment need or even demand for treatment in all women with AUD.

## Conclusion

Although treatment-seeking was not associated with personality traits in this study, women with resolved AUD who had received treatment scored high on several KSP- scales. This suggests that personality assessment can be useful for tailoring individualized treatment programs for women with AUD, including to address guilt proneness, as treatment needs differ between someone whose addiction is associated with high impulsivity and someone with high anxiety and reward-seeking who has adequate impulse control. Still, the foremost clinical importance of our finding is that treatment staff and counsellors are not guided by knowledge of patients personality when detecting persons in need of treatment for alcohol problems, e.g. in PHC-settings, but rather must pay attention to alcohol problems in all patients. Future studies need to explore if women who do not seek treatment have special needs which are not met in usual treatment settings as to attract more women to treatment at an earlier stage of the problem development.

## Abbreviations

AUD - Alcohol Use Disorders. In this study the diagnoses are based on the DSM-III R

AUD Resolved - AUD before the last 12 months prior to interview

AUD Current - AUD during the last 12 months prior to the interview

AUD Lifetime - AUD occurring at any time in life

KSP - Karolinska Scales of Personality

PPV - Positive Predictive Value

PHC - Primary Health Care

SWAG - Screening Women and Alcohol in Göteborg

WHO/ISBRA - World Health Organisation and the International Society for Biomedical Research on Alcoholism

## Competing interests

The author(s) declare that they have no competing interests.

## Authors' contributions

FS planned and created the initial study design.

AÖ drafted the manuscript.

VS contributed substantially in the statistic analysis.

AÖ, GH, AJ and FS, collected data, designed and revised the present paper in cooperation.

The final version of the manuscript has been approved by all the five authors.
